# Living longer, working longer: analysing time trends in working life expectancy in Germany from a health perspective between 2002 and 2018

**DOI:** 10.1007/s10433-022-00707-0

**Published:** 2022-05-23

**Authors:** Chiara Heller, Stefanie Sperlich, Fabian Tetzlaff, Siegfried Geyer, Jelena Epping, Johannes Beller, Juliane Tetzlaff

**Affiliations:** Medical Sociology Unit, Hannover Medical School, Hanover, Germany

**Keywords:** Healthy working life expectancy, Working life expectancy, Self-rated health, Health-related quality of life, Time trend

## Abstract

Population ageing poses growing challenges to social security systems, in particular to public pension funds. The study analyses how Working Life Expectancy (WLE) and Healthy Working Life Expectancy (HWLE) in terms of three health indicators developed in Germany. Based on the German Socio-Economic Panel (GSOEP) from 2002 to 2018 (*n *= 211,141), time trends in labour force rates, mental and physical Health-Related Quality of Life (HRQoL), self-rated health (SRH) and the respective combinations (health indicator*labour force) were analysed for all respondents aged 18–74. WLE and HWLE were calculated using the Sullivan method. WLE and HWLE in men and women at age 18 and 50 clearly increased over time. These increases in HWLE were found in terms of all three health indicators. This development was mainly driven by the clear increase of the labour force rates, since the shares of individuals with good and satisfactory SRH or average and good HRQoL remained largely stable over time. The results show that from a health perspective there have been potentials for increases in WLE during the past two decades and that increasingly more healthy life years are spent economically active. However, life years in the labour force but in poor health have increased, too. The absence of clear improvements in health emphasises the importance of current and future preventive measures to maintain health, especially among the middle-aged and older labour force.

**Supplementary Information:**

The online version contains supplementary material available at 10.1007/s10433-022-00707-0.

## Introduction

Life expectancy has been increasing significantly over the past decades (Eurostat 2020a). Combined with low birth rates, this has driven the ageing of populations, which poses challenges to social security systems, especially the pension system. As a consequence, many countries have raised the statutory retirement age and the debate on the potentials and limits of prolonging working lives increasingly gained public attention (Hytti and Valaste 2009; Anderson et al. 2019). So far, however, research on time trends in working life expectancy and on how much of the additional years in labour are spent in good health is still rare. Although the indicator *“Healthy working life expectancy”* had been proposed already in 2007 (Lievre et al. 2007), not many studies have applied it (Parker et al. 2020a). This study contributes to the current state of research by comparing time trends in the length of working lives and in years spent both in labour and in health in the German population.

The population´s ageing has led to an imbalance between the working and non-working population (European Commission Economic Policy Committee Economic and Financial Affairs 2018). In response, policies have been implemented in recent decades to strengthen labour force participation (LFP), especially in the elderly population, and to prolong working lives (Vaupel and Loichinger 2006; Hofäcker et al. 2019). However, since LFP across the full range of working-age affects the average lengths of working lives, focusing on the developments among the elderly alone could not provide a complete picture. This becomes apparent in view of increasing periods of education and vocational training, which reduce the number of working years at younger ages and thus have an impact on the development of the length of working lives over time. Changes in the duration of working life can be analysed in terms of trends in working life expectancy (WLE). This indicator combines information on mortality and LFP and was developed to depict the expected number of remaining economically active years at a given age (Loichinger and Weber 2016). Over the last decades, WLE increased in most European countries (Weber and Loichinger 2020; Eurostat 2021a). For Germany, Eurostat reported an increase in WLE at age 15 of 3.0 years (to 40.7 years) in men and of 5.0 years (to 36.5 years) in women between 2002 and 2018 (Eurostat 2021b). These increases were mostly driven by the increase in employment among the older population aged 55 and older (Eurostat 2021a). As in other European countries, early retirement schemes in Germany have favoured early labour market exits in the past. However, LFP rates have increased strongly over the last two decades (Brussig 2009) since these regulations have been increasingly rolled back in recent years, leading to a prolongation of working lives. In addition, LFP rates have increased due to rising share of economically active women (Brussig 2009; Eurostat 2021c).

Many studies confirm the strong link between health and early retirement and indicate that older individuals are often forced to leave the labour market prematurely as consequence of health conditions (Wubulihasimu et al. 2015; Wahrendorf et al. 2017; Ilmakunnas and Ilmakunnas 2018). Therefore, previous research interpreted healthy life years as a reservoir to extent working lives (Loichinger and Weber 2016; Weber and Loichinger 2020). Comparing healthy life expectancy and years in labour, studies imply that there is some potential for increasing WLE in several European countries including Germany (Loichinger and Weber 2016; Weber and Loichinger 2020). However, when it comes to potentials for further increases of life years in labour, trends in the length of working life in good health also need to be considered. This holds especially since being in labour but in poor health is strongly associated with poor work ability (Leijten et al. 2014), sick leave (Gustafsson and Marklund 2014), and premature labour market exits (van Rijn et al. 2014; Wahrendorf et al. 2017). Therefore, research started to focus on trends in “healthy working life expectancy” (HWLE) (Lievre et al. 2007), i.e. the expected number of life years an individual spends healthy and in the labour force. A recent study from the UK suggests that HWLE is much lower than the statutory pension age and several years in labour were spend in poor health (Parker et al. 2020b). Other studies even suggest that WLE in poor health have increased (Wind et al. 2018) and years in labour are increasingly accompanied by chronic diseases in many European countries, including Germany (Boissonneault und Rios 2021).

Previous research suggests that subjective health is of great importance for predicting LFP and individual plans concerning the timing of retirement (van Solinge and Henkens 2010; Ilmakunnas and Ilmakunnas 2018). According to the WHO, health is a multidimensional concept, defined as “a state of complete physical, mental and social well-being” (World Health Organization 2020) emphasising the importance of the individual’s perception of the own health and the use of subjective health indicators to depict trends in population health. Among subjective health indicators, self-rated health (SRH) and health-related quality of life (HRQoL) are two of the most commonly used outcomes in studies analysing population health (Guyatt et al. 1993; Macias et al. 2015). SRH represents a global summary measure of self-perceived health, in which the assessment of health depends on various individual factors, such as medical diagnoses, physical limitations, mental conditions and social role perceptions (Garbarski 2016). It reflects how respondents assess their overall health, considering health expectations and their individual health progression (Jylhä 2009). SRH is widely considered a reliable health measure since it is associated with various health outcomes, including specific diseases, functional limitations, health care utilisation, and mortality (De Salvo et al. 2005; Idler et al., 1992; Jylhä 2009, Mavaddat et al. 2014). Quality of life refers to the individual perception of the personal life situation, based on the subjective assessment of positive and negative aspects of life (The WHOQOL Group 1998). HRQoL focuses on those aspects of overall quality of life that affect physical or mental health (Centers for Disease Control and Prevention (CDC) 2000). It depicts health more specific since it allows to discriminate between mental and physical HRQoL in two subscales. Previous research has shown that health has a substantial impact on individual LFP and premature retirement and that it also affects other factors relevant to labour force decisions, e.g. work motivation and work ability (Nilsson et al. 2011; Tisch 2015). In this respect, SRH and HRQoL are of great interest, as they are proven to be associated with WLE (Pedersen andBjorner 2017), work ability (Sörensen et al. 2008), early labour market exists (Tisch 2015; Mäcken 2019), and sick leaves (Gustafsson and Marklund 2014).

With respect to trends in SRH in Germany, studies reported increases in the odds of good SRH especially for the elderly, while trends among younger and middle-aged individuals remained stable or tend to decrease over time (Sperlich et al. 2019, 2020). In sum, it seems to be mainly the positive trend among the elderly that fostered gains in healthy life years over time among the total German population since the increase in the proportion of individuals having good SRH was most pronounced among individuals aged 61–70 years and 71–80 years (Sperlich et al. 2019). Similar results were found for HRQoL. While mean values of physical HRQoL remained rather constant in men and women aged 30–64 years, increases were found in the age group 65 years and older. In contrast, mental HRQoL increased more clearly over time (Klar et al. 2021). For Germany, these trends had increased the share of healthy lifespan since healthy life years rose more than total life expectancy over time (Sperlich et al. 2019; Klar et al. 2021). While life expectancy of women is higher than that of men, the risk of chronic activity limitations is higher among women, thus leading to a gender gap in health expectancies (van Oyen et al. 2010). However, with respect to SRH, this gender gap narrowed in Germany over the past 15 years as women gained more healthy life years than men (Sperlich et al. 2019). Moreover, gender differences in LFP have changed substantially over time. Compared to men, women´s LFP rates has been much lower in the past. As a result of the strong increases in employment rates among women, the gap in LFP rates between genders has been declining (Statistisches Bundesamt 2021b). In 2019, 83.5% of males and 74.9% of females aged 15–65 belonged to the labour force (Statistisches Bundesamt 2020).

Our study aims to examine how working life expectancy (WLE) developed over time and how many years were spent both, economically active and healthy. Furthermore, time trends in LFP and health by gender and age will be investigated. Health is assessed by self-rated health (SRH) and health-related quality of life (HRQoL). Against the backdrop of the considerations above, we aimed to tackle the following research questions:

- How did the working life expectancy (WLE) in the German population develop over time?
- How did the healthy working life expectancy (HWLE) develop over time?
- Did the relation between WLE and HWLE change over time?
- Did these developments differ between women and men?

## Method

### Data

The analyses are based on the population-based survey data of the German Socio-economic Panel (GSOEP V.35) spanning the period 2002–2018. The GSOEP is an annual survey of individuals in private households, conducted by the German Institute for Economic Research. With over 20,000 respondents per year, the GSOEP is one of the largest survey datasets in Europe that provides information on health, employment status and many other socio-economic characteristics (Schupp 2012; Goebel et al. 2019).

In this study, we applied the definition of labour force as proposed by the International Labour Force Organization (ILO) (Benes and Walsh 2018). Since this definition refers to individuals aged between 18 and 74 years, we included all respondents in this age range. Overall, 211,141 respondents (47.4% men and 52.6% women) were included (Table 1). Given the panel structure of the data, individuals often participated in multiple survey waves. In all analyses, cross-sectional weights were used, which are assumed to provide representative samples to allow for statistical conclusions for the total German population. The weighted sample characteristics are presented in Table 1.

**Table 1:** Weighted characteristics of the study population: case numbers and proportions by gender

	Men		Women	
Labour force status				
Employed	70,276	70.3%	65,994	59.4%
Employed, not working	5	< 0.1%	75	< 0.1%
Unemployed	4298	4.3%	4085	3.7%
Not labour force	25,080	25.1%	40,609	36.5%
Missing	363	0.4%	355	0.3%
SRH				
Satisfying or good SRH	84,552	84.5%	90,618	81.6%
Poor SRH	15,304	15.3%	20,226	18.2%
Missing	166	0.2%	275	0.2%
Physical HRQoL				
Average or good physical HRQOL	81,195	81.2%	85,436	76.9%
Poor physical HRQOL	14,177	14.2%	19,077	17.2%
Missing	4650	4.6%	6606	5.9%
Mental HRQoL				
Average or good mental HRQOL	82,228	82.2%	84,286	75.9%
Poor mental HRQOL	13,142	13.1%	20,227	18.2%
Missing	4652	4.7%	6606	5.9%
Survey year				
2002	11,221	11.2%	12,379	11.1%
2004	11,199	11.2%	12,333	11.1%
2006	11,203	11.2%	12,514	11.3%
2008	11,297	11.3%	12,629	11.4%
2010	11,239	11.2%	12,564	11.3%
2012	10,799	10.8%	12,207	11.0%
2014	10,841	10.8%	12,117	10.9%
2016	11,117	11.1%	12,182	11.0%
2018	11,105	11.1%	12,194	11.0%

Self-Rated Health (SRH), Health-Related Quality of Life (HRQoL), weighting was done by using cross-sectional weights to match official population statistics; data: German Socio-Economic Panel (GSOEP) 2002–2018, individuals aged 18–74 years

### Measures

According to the ILO labour force definition, all individuals aged 18–74 years who were either employed, employed but not working (due to parental leave, sickness absence, further education), or unemployed are classified as part of the labour force (European Commission 2016). Participants who had been working in the past 7 days for at least 1 h were considered to be employed. Employed individuals who have not been working because of maternity/ paternity leave or sickness absence were defined employed, but currently not working. Those, who were actively seeking employment and were currently available to start a new job were defined as unemployed.

To depict different aspects of health, we used three different subjective health indicators for our analyses: self-rated health (SRH), mental health-related quality of life (HRQoL) and physical HRQoL. Overall, subjective health measures are proven to be good predictors for a wide variety of health-related outcomes, including mortality (Jylhä 2009; Brown et al. 2015; de Bruin et al. 1996). SRH was measured by asking participants to assess their current health status using a five-point response scale ranging from ‘very good’, ‘good’, ‘satisfactory’, ‘poor’ to ‘bad’. Assuming that at least a satisfactory SRH is required to successfully participate in the labour market, we subsumed the responses into the binary categories poor SRH (‘poor’ and ‘bad’) and satisfactory or good SRH (‘satisfactory’, ‘good’, ‘very good’, further referred to as satisfactory or good SRH). Further, the HRQoL was measured using the SF-12v2 questionnaire (Andersen et al. 2007; Centers for Disease Control and Prevention 2000), which assessed in the GSOEP survey bi-annually. The SF-12 includes 12 items depicting different health aspects. These items were assigned to eight health dimensions and then summarized into two norm-based scores making up two primary quantities of health: the physical health component summary score (PCS) and the mental health component summary score (MCS) with values ranging from 0 to 100. These values were standardized to a national norm (average scores of GSOEP respondents in 2004) with a mean score of 50 and a standard deviation of 10, which quantifies meaningful upward or downward deviations from this national average (Andersen et al. 2007). Following this definition, we classified PCS and MCS with score values less than or equal to 40 as poor mental and physical HRQoL, respectively, while score values above 40 were classified as average or above average HRQoL (further referend to as average or good HRQoL).

### Statistical analyses

**Fig. 1:**
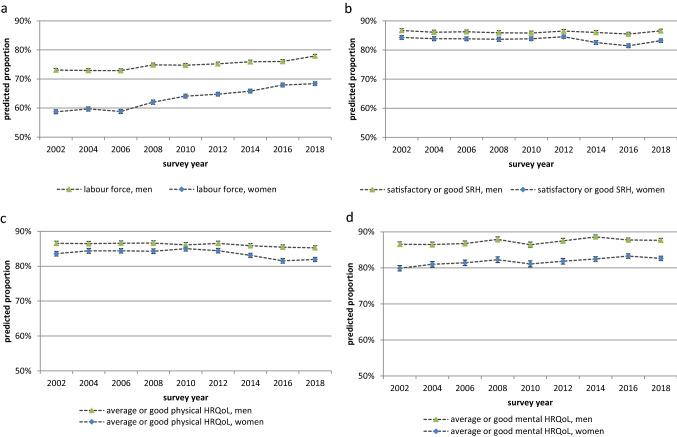
Predicted proportions of **a** labour force participation, **b** satisfactory or good SRH, **c** average or good mental HRQoL, and **d** average or good physical HRQoL from 2002 to 2018 by gender. Self-rated health (SRH), health-related quality of life (HRQoL), predicted proportions are based on logistic regression models which are controlled for age in single-year age groups and include the covariate survey year (categorical), postestimations were performed using the Stata command “margins, pr(pr)”, the model output can be found in S1 95% confidence intervals are based on robust standard errors; data: German Socio-Economic Panel (GSOEP), participants aged 18–74

In order to investigate time trends in LFP and in health, logistic regression models were performed. Each of these models contain survey year (bi-annually as categorical variable) is controlled for age (as single-years) and provides robust estimates for standard errors adjusting for repeated observation of the respondents across survey waves. The models were estimated separately for women and men (Online Resource 1, Table S1). From these models, the overall age-adjusted LFP rates were predicted across survey years for the total of individuals aged 18–74 years. In the same way, the overall proportion of individuals being in satisfactory or good health were estimated for the entire age range 18–74 years. The results are provided in Fig. 1. A deeper insight into the time trends in different age groups has already been published in earlier studies based on the same data basis (Sperlich et al. 2019, 2020; Klar et al. 2021). The results of these studies and their relevance for the present analyses will be discussed.

In order to illustrate which age groups contributed to the changes of WLE over time, we analysed changes in (healthy) LFP across age. As the trajectories across age are quite smooth and do not show strong fluctuations, this was done by calculating the observed proportion of individuals being 1) in the labour force or 2) in the labour force and in satisfactory/average or good health according to the three health outcomes by age group for the years 2002 and 2018.

**Fig. 2:**
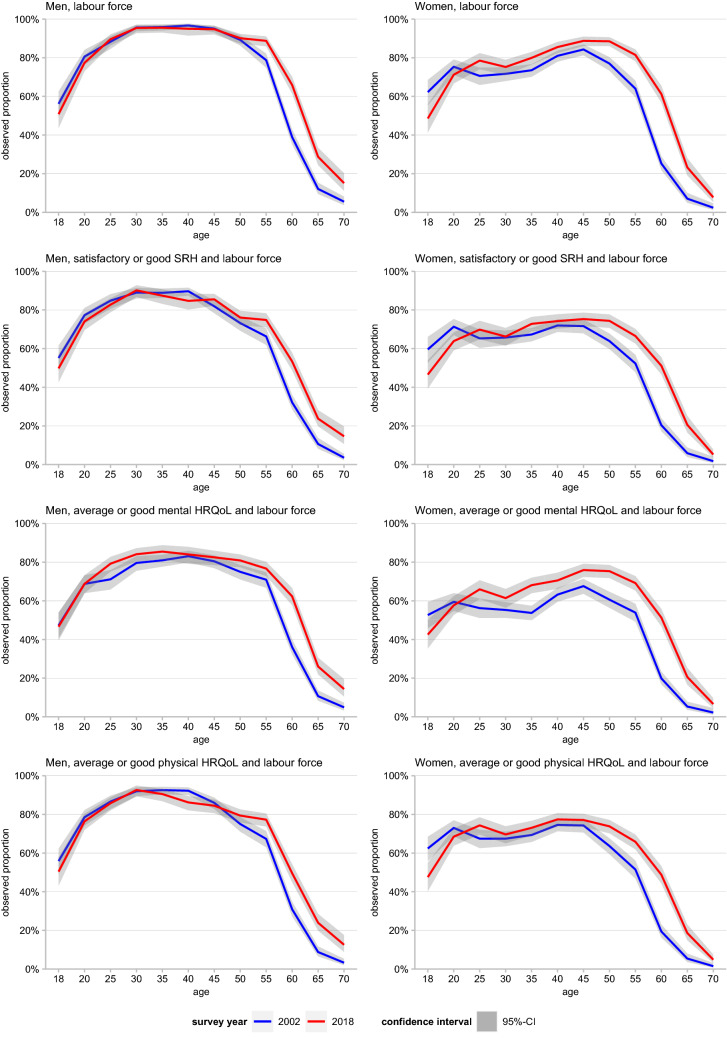
Observed labour force participation rates and of labour force participation rates by health status and gender in 2002 (blue) and 2018 (red). Shadows indicate 95% confidence intervals (CI), CI are based on robust standard errors; Self-Rated Health (SRH), Health-Related Quality of Life (HRQoL) data: German Socio-Economic Panel (GSOEP), participants aged 18–74 years

In order to estimate trends in life years individuals can expect to be 1) part of the labour force and 2) in the labour force and in satisfactory/average or good health, we calculated the working life expectancy (WLE) and the healthy working life expectancy (HWLE) (Lievre et al. 2007) based on the observed values presented in Fig. 2 and S2 (Online Resource 1). While WLE depicts the number of life years an individual can expect to be part of the labour force, HWLE depicts the number of life years expected to spend both, healthy and in the labour force. HWLE thus represents a subset of the WLE, with the difference between the WLE and the HWLE depicting the number of years in the labour force spent in poor health. For these calculations, we applied Sullivan’s method (Jagger et al. 2014) which combines information on life expectancy from period life tables commonly derived from official population statistics and on the age-specific proportions of individuals in the labour force (WLE) and in satisfactory/average or good health (HWLE) derived from the survey data. As a prerequisite of the Sullivan analyses, we calculated partial life tables from age 18 up to the age of 74 years – the upper age limit for LFP according to the ILO definition. The data required on annual age-specific mortality and population size were obtained from the German Federal Statistical Office (Statistisches Bundesamt 2021c, 2021a). Since the GSOEP only contains information on HRQoL every two years, Sullivan life tables were calculated for all even years as well (2002, 2004, …, 2018). To allow for a broader picture in health among the labour force, trends in HWLE were analysed based on the proportion of subjects belonging to the labour force and at the same time do not having more severely impaired health according to the three health indicators: satisfactory and good SRH, average or good physical and mental HRQoL. All analyses above are stratified by gender and performed using Stata 14 MP (Stata Statistical Software 2015).

### Sensitivity analyses

In order to test the robustness of the reported time trends, sensitivity analyses were performed by using an alternative definition of the labour force. The GSOEP includes information on the current official employment status (Schupp 2012). For the alternative definition, individuals which register officially unemployed are considered members of the labour force as well. In contrast to the ILO definition, this also includes those who were not actively looking for a job or were not able to start a new job within two weeks. Therefore, the share of individuals defined to be part of the labour force is somewhat higher than according to the ILO definition. Performing the calculation of WLE, HWLE and logistic regression models, similar time trends were obtained, thus confirming the robustness of the results reported in this study. However, since more individuals are considered to be part of the labour force, the level of WLE lies above those WLE calculated according to the ILO definition (Online Resource 1, Fig. S5). The results of the sensitivity analyses are attached in the appendix (Online Resource 1, Fig. S3-S6).

## Results

From 2002 to 2018, the LFP increased in men from 73.1 to 77.9%, and even more in women (from 58.7 to 68.4%), thus, the gender gap in labour force participation declined constantly (Fig. 1). Overall, the proportion of individuals without severe health impairments remained quite constant for all three health indicators – SRH, physical and mental HRQoL. While the proportions in average and good mental HRQoL slightly increased in both genders, the predicted proportions of average and good physical HRQoL and SRH in women tended to decrease after 2010 (Fig. 1).

With regard to the distribution of employment across age, a shift in labour force particpation = LFP as introduced earlier, towards increasing participation at older ages was found for both genders. For men, increasing LFP was not evident until age 60. In women, the increase in labour force particpation = LFP as introduced earlier, is particularly strong and also evident in younger women above age 25 (Fig. 2). This development is rather continuous over all years (Online Resource 1, Fig. S2). Due to the rather stable trends in health, the development in the proportions of the healthy labour force was mainly determined by the trend in LFP. Increases in healthy labour force were found mainly at higher ages in which LFP increased strongest, too. Across age, a temporary drop in women's proportion of healthy labour force in terms of mental HRQoL was found around age 35–40 (Fig. 2).

**Fig. 3:**
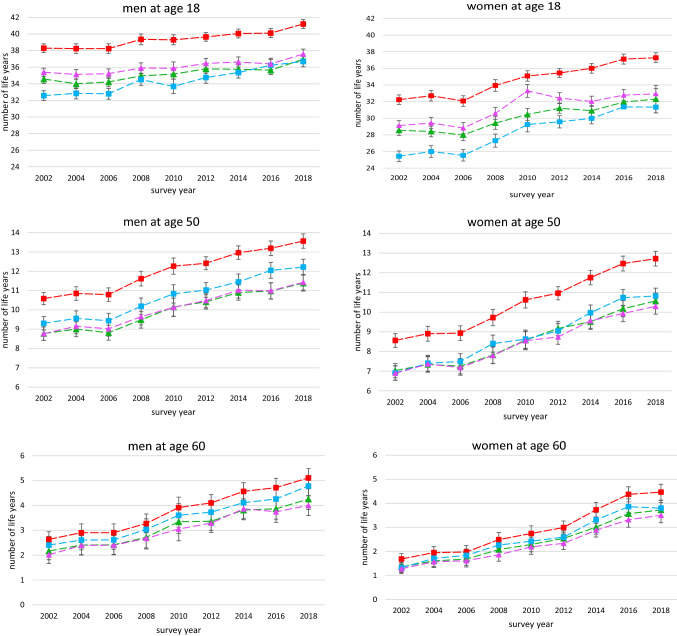
Time trends in Working Life Expectancy (WLE) and Healthy Working Life Expectancy (HWLE) in terms of self-rated health (SRH), mental health-related quality of Life (HRQoL) and physical HRQoL at age 18, 50, and 60 by gender and survey year. 95% confidence intervals are based on robust standard errors; data: German Socio-Economic Panel (GSOEP), participants aged 18–74 years

Over time, WLE clearly increased in both genders (Fig. 3). For women WLE rose particularly strongly from 32.2 to 37.3 years at age 18, from 8.6 to 12.7 years at age 50, and from 1.7 to 4.5 years at age 60. Similarly, WLE also increased among men, albeit more weakly than in women (38.3 to 41.2 years at age 18, 10.6 to 13.6 years at age 50, and 2.6 to 5.1 years at age 60). Mainly driven by the substantial rise in WLE, HWLE also increased over time. These increases were observed for men and women at both younger and older ages. Trends in HWLE at 18 years were weakest with respect to SRH and physical HRQoL in which approximately 2.4 years in men and 3.7 years in women were gained, respectively. In line with growing proportions of subjects in average and good mental HRQoL, increases in HWLE were strongest in terms of mental HRQoL (5.9 years in women and 4.1 years in men at age 18). With respect to the different health indicators, the overall level of HWLE in terms of mental HRQoL at age 18 is significantly lower than the HWLE with respect to SRH and physical HRQoL. Among men at age 50, mental HWLE is higher than those related to the two other health indicators while among women HWLE based on all three health indicators were quite similar in level and development (Fig. 3).

Comparing trends in WLE and in HWLE, the difference between 2002 and 2018 widened in terms of physical HRQoL and SRH. This holds especially for women irrespective of whether age 18 (physical HRQoL: 3.3 to 4.4 years, SRH: 3.7 to 5.0 years), age 50 (physical HRQoL: 1.7 to 2.4 years, SRH: 1.5 to 2.1 years) or age 60 (physical HRQoL: 0.4 to 1.0 years, SRH: 0.3 to 0.7 years) is considered. For men aged 18, the difference also widened, although to a lesser extent (physical HRQoL: 2.9 to 3.6 years, SRH: 3.7 to 4.2 years). These findings indicate that that life years spent in the labour force but in poor health have increased, too (Fig. 3).

**Fig. 4:**
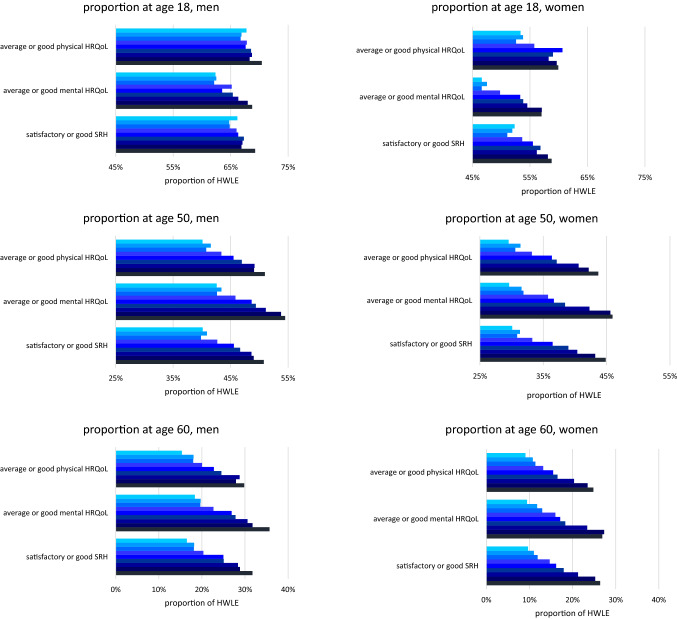
Time trends in the proportion (in %) of Healthy Working Life Expectancy (HWLE) in total life expectancy by health indicator (self-rated health (SRH), mental/physical health-related quality of life (HRQoL)), gender and survey year at age 18, 50, and 60. 95% confidence intervals are based on robust standard errors; data: German Socio-Economic Panel (GSOEP), participants aged 18–74 years

The proportion of HWLE in total life expectancy showed a distinct increase as well. This trend appeared simultaneously for all three health indicators. For example, the proportion of HWLE in terms of SRH at age 18 for women increased from 52.3 to 58.8%, and for men from 66.2 to 69.3%. This increase was even higher at age 50 (30.1 to 44.9% in women and 40.1 to 50.8% in men) and age 60 (9.7 to 26.4% in women and 16.5 to 31.7% in men). Overall, the greatest proportion of HWLE in total life expectancy was observed for physical HRQoL at age 18 and for mental HRQoL at age 50 and above (Fig. 4).

## Discussion

Our study shows that WLE increased substantially at age 18 as well as at higher working age. This development is mainly due to increased LFP among women and men at older ages. Since this increase was generally stronger in women and was also apparent at younger ages, the gender gap in WLE reduced over time. HWLE has been rising as well, which holds true for all three health indicators. This trend is apparent in both the growing absolute numbers of healthy working life years and as relative proportion in total life expectancy. However, the increase was primarily driven by increasing LFP and not due improving health since proportions of individuals without more severely impaired health remained largely stable. Comparing HWLE and WLE, the difference has widened especially for women in terms of SRH and physical HRQoL, indicating that economically active years of life spent in poor health have increased, too. Among men, this development is also apparent, though differences widened at a slower pace.

### Trends in HWLE and WLE

Previous studies have shown that WLE has been increasing over time in almost all European countries (Loichinger and Weber 2016; Weber and Loichinger 2020). For Germany, an increasing WLE was reported for the younger (Eurostat 2021b) as well as for the older working-age population (Loichinger and Weber 2016; Weber and Loichinger 2020; Dudel et al. 2021) Consistent with this, we also found increasing WLE over time in both genders among the German population. LFP increased strongest in women and above the age of 50 years in both genders, which have reduced the gender gap in WLE over time. Similar results were also obtained in other studies which highlights the importance of later retirement and the higher proportion of women in the labour force as the primary causes for rising WLE (Brussig 2009; Weber and Loichinger 2020). Compared with other European countries, the increases in WLE of men in Germany was rather moderate. This might be due to the fact that WLE of men had already reached a comparably high level (Loichinger and Weber 2016). Women's WLE, on the other hand, was much lower and thus had more potential to increase. Since LFP among German women has increased over a wide age range, a particularly strong increase in WLE was observed (Loichinger and Weber 2016).

Although a positive correlation between healthy life years and WLE could be demonstrated in earlier studies (Pedersen and Bjorner 2017; Weber and Loichinger 2020), our findings also indicate increasing LFP to be by far the greater driver in HWLE than trends in health. This indicates that increasing shares of healthy life years were spent in labour over time and that with respect to SRH and HRQoL there had been much potential to extent working lives in the past. This potential was favoured by the trend towards improving health in the past, especially among the elderly and in terms of subjective health indicators, leading to gains in healthy life years and to morbidity compression (Sperlich et al. 2019; Klar et al. 2021). The findings are also in-line with the results of Weber and Loichinger, which also show that the number of expected healthy life years is above the WLE but the difference decreased over time (Loichinger and Weber 2016; Weber and Loichinger 2020). Recent analyses by Boissonneault and Rios show that the increase in WLE was accompanied by rather constant HWLE and major increases in years in labour spent with chronic diseases in Germany. The lack of positive trends in HWLE may be at least partly due to the broad definition of health as the absence of a range of chronic diseases. However, not all of these diseases are necessarily associated with impaired ability to work. Applying a more rigorous definition of poor health, HWLE was also found to increase (Boissonneault and Rios 2021).

However, due to the lack of a clear trend in the three health outcomes towards improving health, it remains to be seen whether the increase in HWLE will continue in the future. Considering the lifespan free of specific diseases often fostering early labour market exists, the evidence is limited and provides a mixed picture. While previous studies reported increases in the lifespan without severe cardiovascular events, such as stroke and myocardial infarction (Geyer et al. 2018; Tetzlaff et al. 2020a), and lung cancer (Tetzlaff et al. 2020b), the number of life years free of type 2 diabetes and multimorbidity decreased over time (Muschik et al. 2017; Tetzlaff et al. 2017). Moreover previous studies have shown that trends in subjective health differ according to age group: For above-average physical and mental HRQoL as well as for good and very good SRH, increasing life years in good health were reported for both genders (Sperlich et al. 2019; Klar et al. 2021). However, while improving health was reported among the elderly, constant or worsening trends in SRH and HRQoL were observed for younger age groups (Sperlich et al. 2019, 2020; Klar et al. 2021). Moreover, previous research has shown that work environment-related physical and psychosocial risk factors have a substantial effect on various health outcomes and health inequalities among the working population (e.g. Nieuwenhuijsen et al. 2010; Hoven and Siegrist 2013; Theorell et al. 2016). Given the importance of work-related factors, more research is needed in order to investigate the effect of work environment and work conditions on the development of HWLE more deeply. This may be achieved by identifying developments in the work environment and working conditions that affect health trends and by quantifying their contribution to increases or decreases in HWLE.

### Strengths and limitations

To our knowledge, this study is one of the very few to calculate time trends in HWLE for Germany and the first investigating different subjective health outcomes. The study is based on the large sample of the GSOEP that includes detailed employment-related information and therefore enables the identification of the labour force status according to the widely used ILO definition. In addition, the data contain a lot of information on the health of the respondents, which allowed us to analyse differentiated trends in the labour force according to different health outcomes and thus gain a deeper insight into the development of HWLE.

As with other survey samples, the proportion of individuals in good health is likely to be overestimated as institutionalized individuals and individuals who could not participate in the survey for health reasons are not included. Therefore, the level of HWLE calculated in this study may be higher than in the general population. Nevertheless, the reported trends in health and HWLE remain unaffected as long as this health-response bias did not change over time. Despite the comparatively high response rate in the SOEP (75.3% overall response rate at the individual level and 83.8% at the household level in 2018) (Bohlender et al. 2018), it cannot be completely ruled out, however, that selectivity due to non-response might have influenced the level of WLE in this study. Compared to the "duration of working life" reported by Eurostat, the WLE in our study tends to be somewhat higher (Eurostat 2021b) due to higher LFP rates in the GSOEP (Online Resource 1, Figure S7). The WLE reported by Eurostat is based on the EU-Labour Force Survey, for which participation is mandatory in Germany. These differences might therefore be due to differing response rates that may have increased the share of economically active respondents in the GSOEP data. Despite level differences in WLE, we consider the data appropriate for analysing time trends in (H)WLE, since the development of WLE in our study hardly differs from the time trends reported by Eurostat (Eurostat 2021b). Moreover, individuals with very low and very high socio-economic status (SES) may be underrepresented as well, which however, has been addressed by weighting the data to adjust for differences in SES structure between the survey and the general population.

Previous studies based on the same dataset have demonstrated that SRH as well as HRQoL improved, but that these improvements were mainly driven by the strong increases in the proportion of individuals with good health in their sixties and older. The studies suggest that the overall positive trend towards gains in healthy life expectancy is mainly driven by the elderly, but not necessarily by the younger age groups (Sperlich et al. 2020, 2019; Klar et al. 2021). As our study focuses on the working-age population aged 18–74 years, rather stable trends in SRH and physical HRQoL were found, since the positive developments among the elderly were partly not captured.

As Sullivan’s method represents a period measure and refers to the prevalence of a respective year, no conclusions for future trends can be derived from these analyses. This holds especially since previous research has shown that trends in health differ with respect to age and that younger age groups may show less favourable trends than the older ones (Sperlich et al. 2019, 2020; Klar et al. 2021). However, it has been shown that Sullivan’s method provides good estimates as long as the incidence does not fluctuate considerably over time (Jagger et al. 2014). Since the ILO labour force definition is based on the employment status during a given reference week and since the data contain no information on the time of “incidence” of poor HRQoL and SRH Sullivan’s method was used in this study. However, as our analyses are based on the labour force status during a specific reference week and the health status at the time of interview, we assume that sudden changes in (healthy) LFP would also have been clearly visible in our data, which, however, was not the case (Fig. 2, S2).

With respect to the calculation of HWLE, the results may vary depending on the chosen definition of labour force. Therefore, we conducted sensitivity analyses to test for the robustness of the reported time trends using a second definition. According to this, all individuals who reported to be officially registered as unemployed were assigned to the labour force as well, irrespective of whether they are actively seeking for a job or able to start a new job in the short run. Due to this, the proportion of labour force is higher than according to the ILO definition. Consequently, the general level of WLE and HWLE is higher, while time trends remain stable (Online Resource 1, Fig. S3 and S5).

This study concentrates on health and labour force trends in general, as the official population statistics do not provide mortality data by SES. Therefore, the official life expectancy of the German population on which the calculations of (H)WLE are based was not available by socio-economic characteristics. However, previous studies have shown that both overall life expectancy (Tetzlaff et al. 2020c) and disease-free life years in Germany differ substantially between SES groups (Tetzlaff et al. 2020a, 2020b, 2021). Given the profound effect of SES on health and employment, it remains open to whether the outlined trends among the German population vary by SES. Therefore, further research should concentrate on whether all SES groups benefitted equally from increasing HWLE and to what extent life years spent in labour but in poor health differ whenever the data allow for such analyses. Furthermore, it has to be kept in mind that the decision to leaving the labour market prematurely and to stay in labour beyond the statutory retirement age is not based solely on health-related considerations. Previous research also suggests financial reasons to play an important role on the age of retirement in Germany. Hence, the choice of retirement timing also depends on individual socio-economic characteristics and is therefore socially unequally distributed (Hess 2018). This emphasizes that trends and socio-economic differences in WLE and their underlying causes should be investigated in more detail in future studies.

## Conclusion

Our study showed substantial increases in WLE as well as in HWLE over time. This development was primarily due to an increase in labour force among older individuals and females, while health remained largely stable over time. Consequently, healthy life years were increasingly spent economically active. Moreover, the share of HWLE in total life expectancy increased, indicating that years spent in the labour force and in good health increased stronger than total life expectancy. However, due to the absence of clear improvements in health among the working-age population the gap between HWLE and WLE tended to narrow over time and it remains open whether and to what extent the HWLE will continue to rise in the future. Whether the reported trends can be applied to all SES groups requires further investigation. In order to understand the influence of different job exposures, further studies should analyse the link between different job exposures and health as well as its effects on the development of WLE over time.

## Supplementary Information

Below is the link to the electronic supplementary material.

Supplementary file1 (PDF 2543 kb)

## Data Availability

The raw data were drawn from the German Socio-Economic Panel Study carried out at DIW (Deutsches Institut für Wirtschaftsforschung) (GSOEP V.35). The datasets used are available from the corresponding author on reasonable request. German data privacy laws necessitate that all users sign a data user contract with the DIW.
