# Supplementary material for: Living longer, working longer: analysing time trends in working life expectancy in Germany from a health perspective between 2002 and 2018
Source: Eur J Ageing. 2022 May 23;19(4):1263–76. doi: 10.1007/s10433-022-00707-0 (PMC9729498; doi:10.1007/s10433-022-00707-0)
Supplement: Supplementary file 1 — Supplementary file1 (PDF 2543 kb) [file 10433_2022_707_MOESM1_ESM.pdf]

European Journal of Ageing

Online Resource 1

Living longer, working longer: Analysing time trends in working life expectancy in Germany from a health perspective between 2002 and 2018

Chiara Heller[1], Stefanie Sperlich[1], Fabian Tetzlaff[1], Siegfried Geyer[1], Jelena Epping[1], Johannes Beller[1], Juliane Tetzlaff[1*]

[1] Medical Sociology Unit, Hannover Medical School, Hanover, Germany

*Corresponding author

E-mail: tetzlaff.juliane@mh-hannover.de

Table S1 Time trends in SRH, mental HRQoL and physical HRQoL by gender

	**SRH**[1]		**Mental HRQoL**[2]		**Physical HRQoL**[2]	
	OR	95%-CI	OR	95%-CI	OR	95%-CI
**Men**						
Age	0,94	0,94-0,94	0,95	0,95-0,96	0,93	0,93-0,94
Survey year						
2004	0,93	0,88-0,99	1,01	0,94-1,08	0,96	0,90-1,03
2006	0,96	0,89-1,02	1,01	0,94-1,08	0,97	0,90-1,05
2008	1,0	0,92-1,08	1,16	1,07-1,26	1,02	0,92-1,11
2010	1,04	0,95-1,14	1,07	0,98-1,18	1,01	0,92-1,11
2012	1,13	1,04-1,22	1,20	1,10-1,30	1,10	1,01-1,19
2014	1,12	1,03-1,22	1,28	1,18-1,40	1,13	1,03-1,23
2016	1,12	1,02-1,23	1,42	1,29-1,56	1,11	1,01-1,22
2018	1,26	1,16-1,38	1,46	1,33-1,59	1,26	1,15-1,37
**Women**						
Age	0,94	0,94-0,94	0,96	0,95-0,96	0,93	0,93-0,94
Survey year						
2004	0,98	0,92-1,04	1,08	1,01-1,15	1,02	0,96-1,09
2006	0,98	0,95-1,05	1,07	1,00-1,15	1,01	0,94-1,09
2008	1,07	0,99-1,15	1,19	1,10-1,28	1,12	1,04-1,21
2010	1,15	1,07-1,25	1,25	1,15-1,36	1,25	1,15-1,37
2012	1,25	1,16-1,34	1,37	1,27-1,48	1,28	1,18-1,39
2014	1,27	1,18-1,37	1,53	1,42-1,65	1,34	1,24-1,45
2016	1,41	1,30-1,53	1,77	1,63-1,92	1,46	1,34-1,59
2018	1,47	1,36-1,59	1,77	1,63-1,91	1,49	1,38-1,62

[1] satisfactory or good

[2] average or above

Note: SRH self-rated health, HRQoL health-related quality of life, odds ratios (OR) of satisfactory or good SRH/ average or above mental and physical HRQoL were estimated based on binary logistic regression models. The models are stratified for gender and include the covariates age in single-year age groups (continuous variable) and survey year (categorical). 95% confidence intervals are based on robust standard errors; data: German Socio-Economic Panel (GSOEP), participants aged 18 to 74 years

Figure S2 Observed proportions of labour force participation and of labour force participation by health status and gender for all even survey years from 2002 (dark blue) to 2018 (dark red)

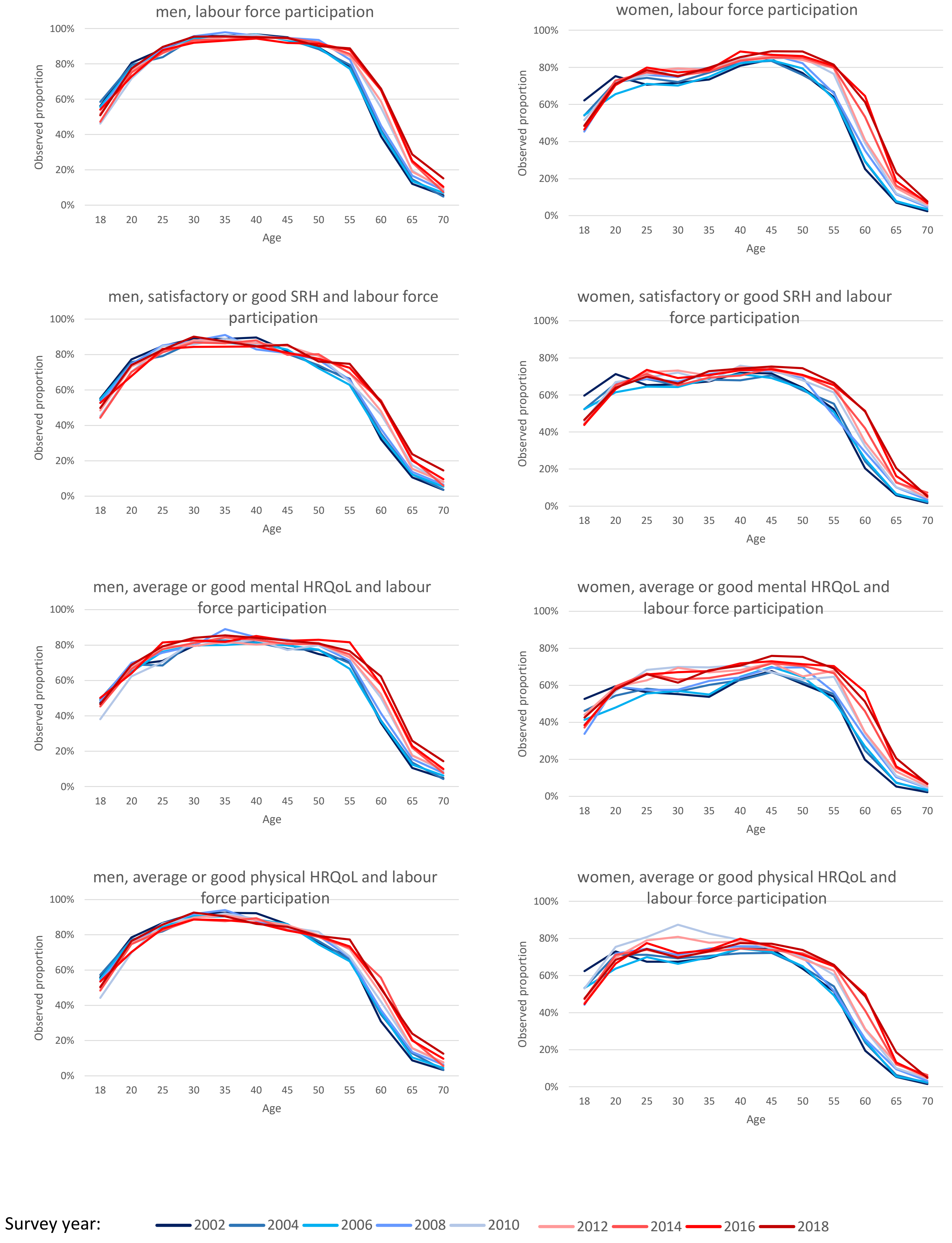

Figure S3 Predicted proportions of labour force participation from 2002 to 2018 by gender

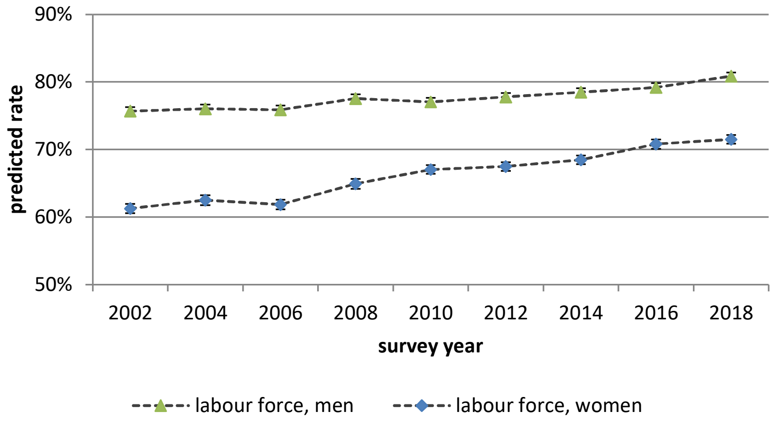

Note: predicted proportions are based on logistic regression models which are controlled for age in single-year age groups and include the covariate survey year (categorical), postestimations were performed using the Stata command “margins, pr(pr)”, 95% confidence intervals are based on robust standard errors; data: German Socio-Economic Panel (GSOEP), participants aged 18 to 74 years

Figure S4 Observed labour force participation rates and of labour force participation rates by health status and gender in 2002 (blue) and 2018 (red)

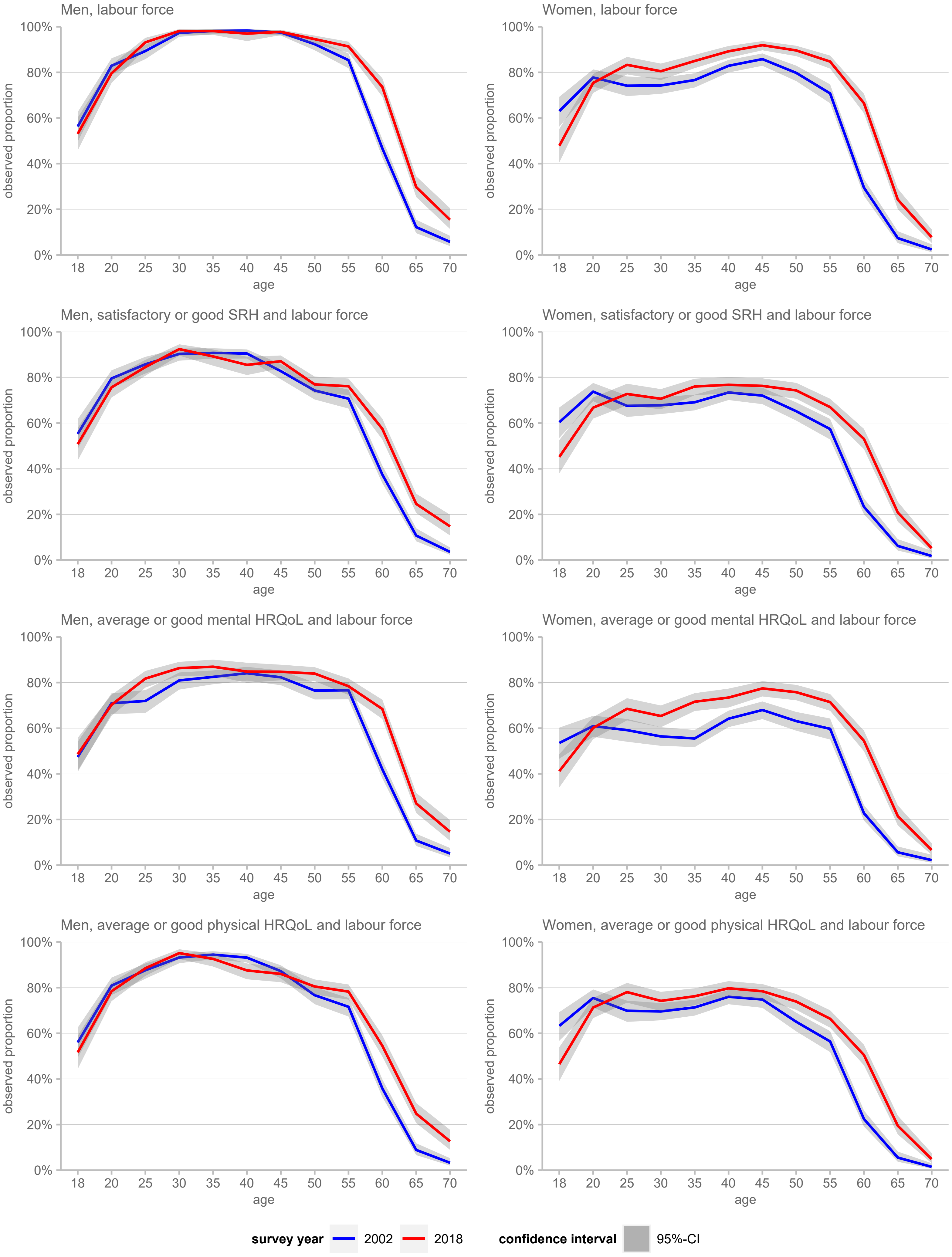

Note: shadows indicate 95% confidence intervals (CI), CI are based on robust standard errors; Self-Rated Health (SRH), Health-Related Quality of Life (HRQoL)

Data: German Socio-Economic Panel (GSOEP_V35), participants aged 18 to 74 years

Figure S5 Time trends in Working Life Expectancy (WLE) and Healthy Working Life Expectancy (HWLE) in terms of self-rated health (SRH), mental health-related quality of Life (HRQoL) and physical HRQoL at age 18, 50, and 60 by gender and survey year

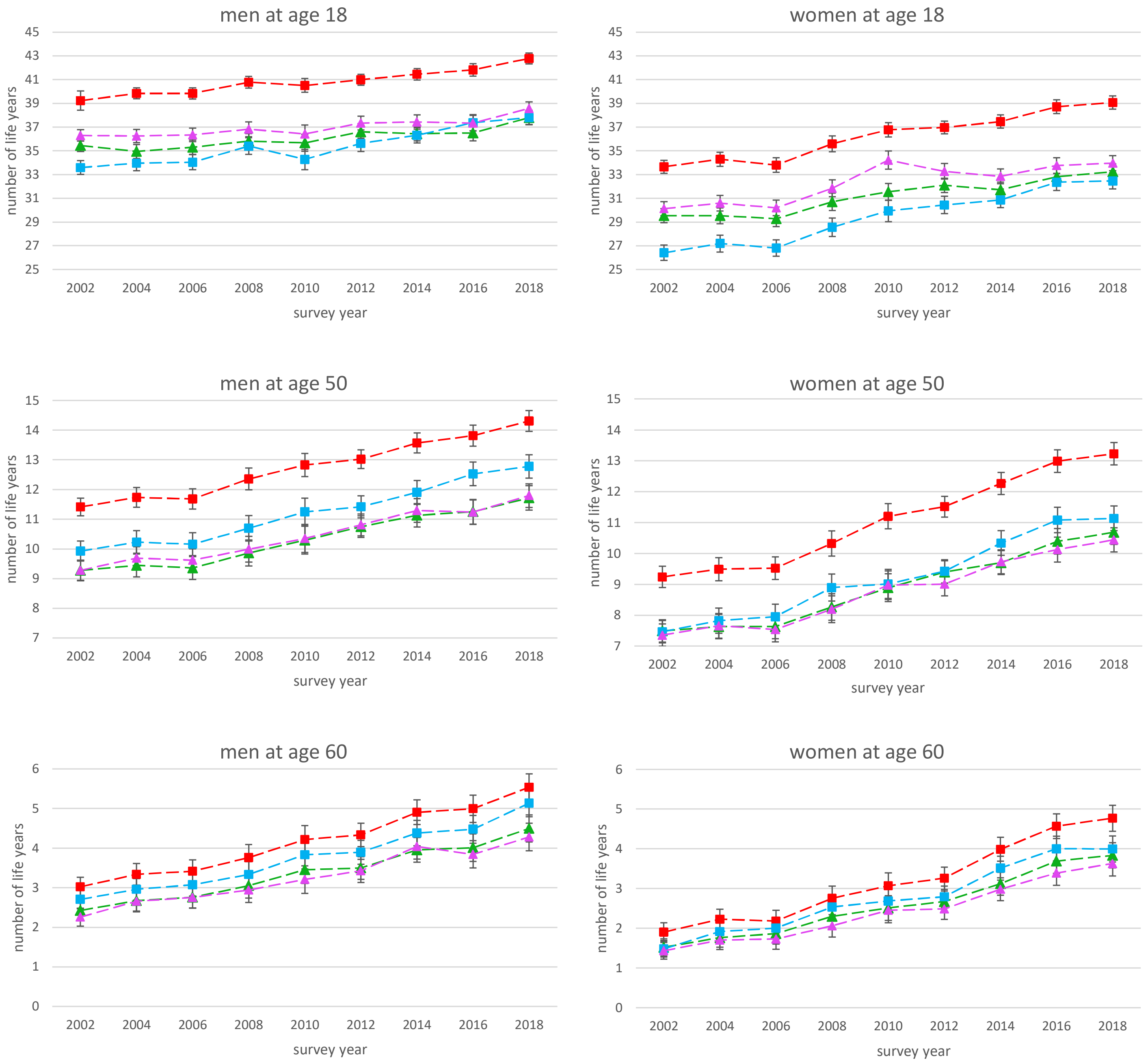

Note: 95% confidence intervals are based on robust standard errors; data: German Socio-Economic Panel (GSOEP), participants aged 18 to 74 years

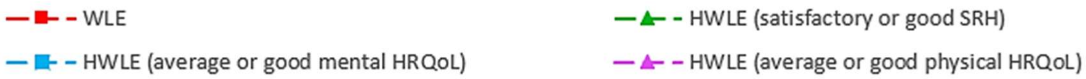

Figure S6 Time trends in the proportion (in %) of Healthy Working Life Expectancy (HWLE) in total life expectancy by health indicator (self-rated health (SRH), mental/physical health-related quality of life (HRQoL)), gender and survey year at age 18, 50, and 60

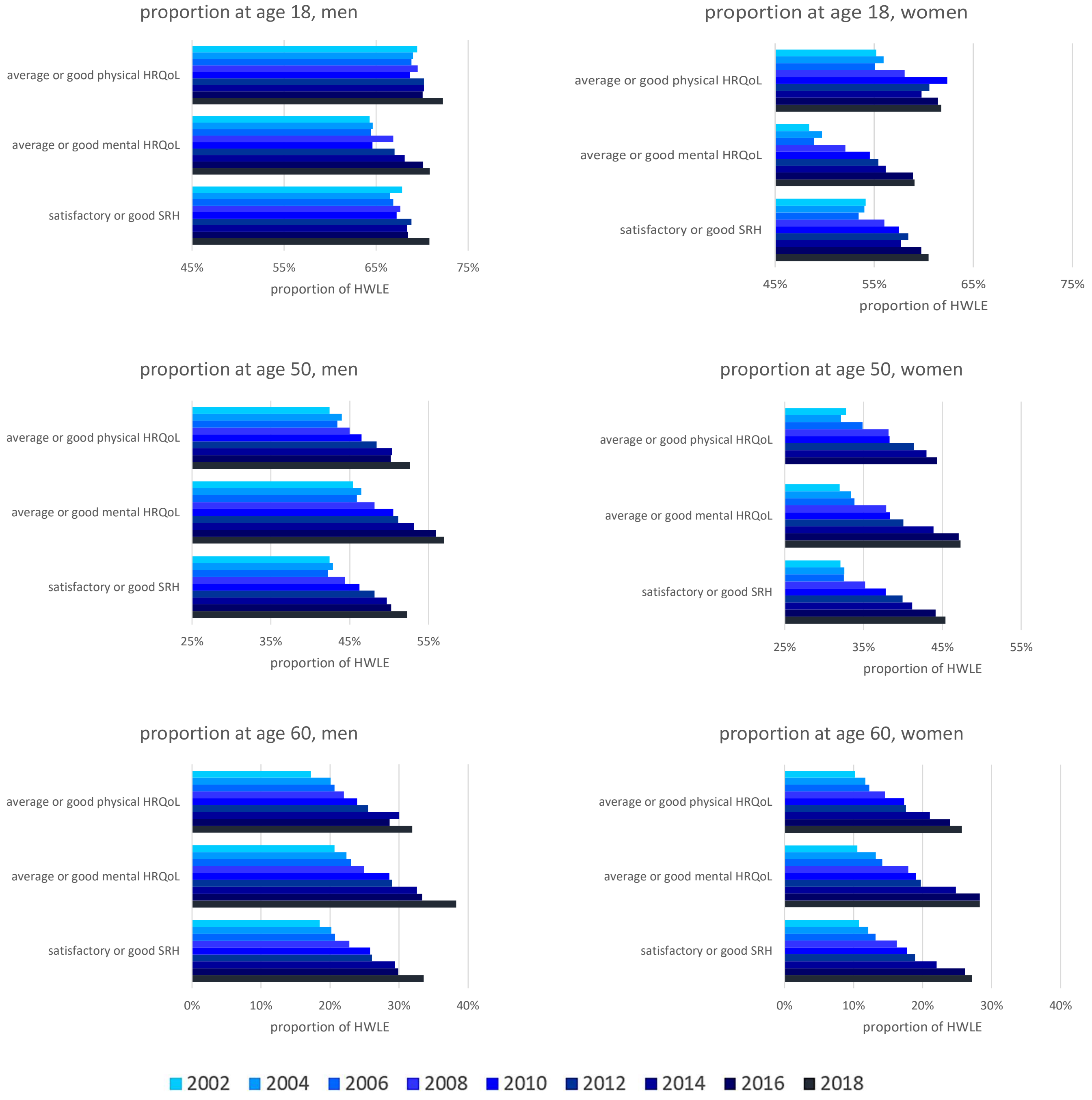

Note: 95% confidence intervals are based on robust standard errors; data: German Socio-Economic Panel (GSOEP), participants aged 18 to 74 years

Figure S7 Age-specific labour force rates (in %) based on the GSOEP and Eurostat by gender and survey year

Men	2002		2004		2006		2008		2010		2012		2014		2016		2018	
	GSOEP	Eurostat	GSOEP	Eurostat	GSOEP	Eurostat	GSOEP	Eurostat	GSOEP	Eurostat	GSOEP	Eurostat	GSOEP	Eurostat	GSOEP	Eurostat	GSOEP	Eurostat
20-24	80.6	73.9	79.4	71.4	77.8	73.5	77.1	74.1	71.7	72.5	76.4	72.1	75.1	70.9	72.9	69.6	77.4	71.5
25-29	88.3	86.0	83.8	84.0	86.8	86.5	88.6	86.9	87.9	87.2	86.0	87.2	86.2	86.4	87.7	85.6	89.6	87.1
30-34	95.6	95.1	94.6	94.5	94.1	95.5	95.6	94.7	93.1	94.6	93.0	94.4	93.5	94.1	91.9	92.7	95.4	92.9
35-39	95.8	95.7	96.0	95.9	95.0	96.6	97.9	96.2	95.6	95.7	95.0	95.6	93.5	95.0	93.1	94.0	95.5	94.3
40-44	96.7	95.2	95.5	95.8	96.8	96.0	95.8	95.8	96.4	95.6	94.9	95.3	95.2	95.1	94.3	93.7	95.0	94.0
45-49	95.0	94.3	94.1	94.2	93.3	94.6	94.6	94.5	94.0	94.1	94.3	94.0	94.7	93.7	91.8	93.8	94.7	93.6
50-54	89.2	90.5	88.3	89.9	89.1	91.5	93.5	91.2	92.2	91.0	91.5	91.7	92.1	91.3	91.4	91.9	90.1	92.2
55-59	78.7	77.3	79.5	80.3	77.2	82.2	82.4	83.6	84.6	84.9	84.3	85.8	85.6	86.3	87.9	87.4	88.7	87.4
60-64	38.9	33.3	42.4	37.8	41.5	42.5	45.0	46.8	55.1	53.8	58.7	59.0	65.1	63.3	65.0	64.6	65.9	67.9
65-69	12.1	7.0	14.6	7.2	13.6	8.5	16.7	10.0	19.5	10.9	18.9	14.6	24.2	17.8	25.1	19.5	28.7	21.2
69-74	5.5	3.8	4.9	3.7	7.0	4.2	8.4	5.1	8.9	5.0	9.8	7.1	7.5	8.0	10.4	9.3	15.1	10.4

Women	2002		2004		2006		2008		2010		2012		2014		2016		2018	
	GSOEP	Eurostat	GSOEP	Eurostat	GSOEP	Eurostat	GSOEP	Eurostat	GSOEP	Eurostat	GSOEP	Eurostat	GSOEP	Eurostat	GSOEP	Eurostat	GSOEP	Eurostat
20-24	75.3	67.0	72.1	63.9	65.6	67.2	72.5	68.6	71.8	67.3	70.9	66.8	72.9	67.1	70.5	66.5	71.2	67.8
25-29	70.6	74.9	74.4	73.8	71.1	75.7	75.9	76.3	76.0	77.6	78.0	78.7	77.3	79.1	79.9	79.1	78.5	79.7
30-34	71.7	76.9	72.1	77.0	70.2	77.1	74.8	76.4	78.7	77.9	79.5	79.6	75.5	80.0	77.3	79.4	75.2	79.8
35-39	73.5	78.5	77.1	78.9	74.9	79.9	79.8	80.1	79.7	80.0	78.7	80.9	77.9	81.1	78.6	80.6	79.9	81.4
40-44	81.0	81.7	82.6	82.0	82.3	84.3	83.2	83.7	84.9	84.3	84.0	84.9	83.6	84.4	88.7	84.8	85.5	84.7
45-49	84.3	81.2	83.5	82.1	83.9	83.6	86.9	84.0	84.8	84.8	86.3	85.4	85.3	85.6	86.8	86.7	88.7	86.4
50-54	77.0	73.6	75.9	76.6	79.4	78.8	82.3	79.8	85.1	81.1	84.0	82.0	85.1	83.1	86.1	83.7	88.5	84.4
55-59	64.0	57.9	66.7	61.3	63.1	65.7	66.1	67.6	76.5	70.5	79.6	73.5	79.9	75.8	80.8	77.3	81.4	78.5
60-64	25.3	15.8	29.1	19.5	29.5	24.4	35.1	29.4	39.2	35.6	40.8	41.2	53.3	48.8	64.6	52.9	61.2	57.1
65-69	7.1	3.9	7.5	4.1	7.8	5.0	11.6	5.6	12.1	6.6	15.0	8.3	16.3	10.5	18.6	12.0	23.3	13.3
69-74	2.4	1.7	3.5	1.5	3.3	1.9	4.7	2.4	5.4	2.4	6.1	3.5	7.5	4.1	6.9	4.3	7.8	5.3

Note: labour force rates were obtained from the German Socio-Economic Panel (GSOEP, V.35) and Eurostat (lfsq_argan https://ec.europa.eu/eurostat/databrowser/view/LFSA_ARGAN__custom_2364103/default/table?lang=en); age group 18-20 was not displayed since the rates from Eurostat are not available
